# Exerkine-loaded exosomes in muscle aging: a nexus of exercise, regeneration, and crosstalk

**DOI:** 10.3389/fcell.2026.1706977

**Published:** 2026-02-27

**Authors:** Yang Li, Qingzhong Wu, Junmin Wang, Jiahao Ding, Jinpeng He

**Affiliations:** 1 College of P. E and Sports, Beijing Normal University, Beijing, China; 2 College of Physical Education, Zhangjiakou University, Zhangjiakou, China; 3 China Football College, Beijing Sports University, Beijing, China; 4 School of Sports and Health Engineering, Hebei University of Engineering, Handan, China

**Keywords:** exercise, exerkines, exosomes, muscle aging, regeneration

## Abstract

This review examines the critical role of extracellular vesicles, specifically exosomes, as mediators of intercellular and inter-organ communication in the context of skeletal muscle aging and regeneration. Skeletal muscle, traditionally viewed as a simple contractile tissue, is now recognized as a potent endocrine organ that secretes a diverse array of signaling molecules, collectively termed “exerkines,” in response to physical activity. We integrate contemporary evidence demonstrating how exercise modulates the release and molecular composition of muscle-derived exosomes, which in turn influence key cellular processes. The report details how exosomal cargo, including non-coding RNAs and proteins, regulates muscle stem cell activation and differentiation, counteracts age-related decline (sarcopenia) by modulating protein homeostasis and inflammation, and facilitates systemic metabolic crosstalk with distant tissues such as adipose tissue. We also critically discuss the burgeoning therapeutic potential of engineered exosomes for musculoskeletal health, while highlighting significant and interconnected challenges in the field, including the lack of standardized methodologies and regulatory frameworks. This review provides a nuanced perspective on the “exerkine” hypothesis, underscoring the potential of exercise-modulated exosomes as both diagnostic biomarkers and novel therapeutic agents for maintaining lifelong muscle health.

## Introduction

Skeletal muscle is a highly plastic and dynamic tissue, essential not only for locomotion and metabolism but also for systemic endocrine regulation (Trovato et al., 2019; Pratesi et al., 2013). Its remarkable adaptability to stimuli such as exercise, injury, and disuse is paramount to maintaining physical function and metabolic health throughout the lifespan (Aversa et al., 2019; Harridge and Lazarus, 2017). However, this adaptability diminishes with age, leading to a progressive and multifactorial syndrome known as sarcopenia—the gradual loss of muscle mass, strength, and function (Aversa et al., 2019; Narici and Maffulli, 2010). Sarcopenia is characterized by a complex interplay of molecular and cellular deficits, including impaired regenerative capacity, mitochondrial dysfunction, chronic inflammation, and a widening “molecular scissors gap” that favors protein degradation over synthesis (Damanti et al., 2025; Tu et al., 2025). The regenerative process itself, particularly following acute injury or chronic atrophy, is fundamentally dependent on the intricate coordination of various cell types, with muscle satellite cells (SCs) serving as the principal, non-redundant stem cell population (Cao et al., 2024; Morisi, 2016). In recent years, a paradigm shift has occurred in the understanding of intercellular communication, moving beyond soluble factors (e.g., myokines) to embrace the critical role of extracellular vesicles (EVs) (Safdar and Tarnopolsky, 2018; Trovato et al., 2019). Among these, exosomes, small lipid-bilayer vesicles (30–150 nm) derived from the endosomal pathway, have emerged as powerful mediators of both localized and long-distance cellular crosstalk (Szatanek et al., 2017; Charreau, 2021). These nanovesicles, which contain a diverse and specific cargo of proteins, lipids, and nucleic acids, are increasingly recognized as “fingerprints” of their originating cells, reflecting their metabolic and physiological state (Gamez et al., 2025; Iizuka et al., 2014; Bhowmik et al., 2025). The confluence of these fields—exercise physiology, exosome biology, and muscle pathology—has given rise to the “exerkine” hypothesis, which posits that the systemic benefits of exercise are, in part, mediated by the modulation of exosomal cargo (Safdar and Tarnopolsky, 2018; Safdar et al., 2016). This review will integrates the current evidence supporting this hypothesis, exploring the mechanisms by which exercise-induced exosomes influence muscle health, detailing their role in inter-tissue communication, and critically evaluating their potential as therapeutic tools and biomarkers. Importantly, the circulating EV pool induced by exercise is heterogeneous and originates from multiple tissues and cell types (e.g., skeletal muscle, adipose tissue, endothelium, immune cells, platelets), each contributing distinct cargo signatures and biological effects (Nederveen et al., 2021). Moreover, the physiological impact of a given exosome is not determined solely by its source cargo, but also by the recipient tissue’s state (e.g., aging, inflammation, insulin resistance), which shapes uptake, signaling competence, and downstream transcriptional responses (Nederveen et al., 2021). In the sections below, we therefore emphasize (i) source- and modality-dependent cargo selection, (ii) multi-organ contributions to the exercise-EV pool, and (iii) recipient-niche dependence as a key determinant of whether EV signaling is adaptive or maladaptive.

### Exosomes: the vehicles of intercellular communication

Exosomes are a subclass of small extracellular vesicles (EVs), typically ranging from 30 to 150 nm in diameter, that originate from the endosomal pathway (Szatanek et al., 2017). Their biogenesis involves the sequential invagination of the plasma membrane, formation of multivesicular bodies (MVBs), and subsequent fusion of MVBs with the plasma membrane to release intraluminal vesicles (ILVs), or exosomes, into the extracellular space (Gamez et al., 2025; Piper and Katzmann, 2007). This process is regulated by specific cellular machinery, including the Endosomal Sorting Complex Required for Transport (ESCRT) family proteins such as ALIX and TSG101, which are often used as canonical markers for exosome identification (Cao et al., 2024; Xie et al., 2022). Exosomes serve as “nano-sized vesicles that serve as mediators for intercellular communication” by delivering a diverse cargo to neighboring or distant cells (Luo et al., 2024; Li et al., 2018). This cargo includes proteins, lipids, and nucleic acids such as messenger RNAs (mRNAs), microRNAs (miRNAs), and other non-coding RNAs (ncRNAs) (Gamez et al., 2025; Santosh et al., 2015). The composition of an exosome’s cargo is not random; it is highly specific and “considered as a reflection of its originating cell” and its physiological or pathological state (Villarreal-Gómez et al., 2025). This is a fundamental principle that underpins their utility as both biomarkers and therapeutic delivery vehicles. The selective packaging of miRNAs is a key aspect of this function, allowing exosomes to modulate the expression of target genes and influence the functionality of recipient cells (Cao et al., 2024; Kučuk et al., 2021).

An early, albeit now largely superseded, hypothesis proposed that exosomes were merely a mechanism for cells to discard “garbage” or excess constituents to maintain homeostasis (Luo et al., 2024; Bauer, 2023). However, this perspective has evolved. The contemporary understanding views them as a targeted, mechanism-driven system for accumulating and delivering specific cellular components, suggesting a purposeful role in regulating intercellular communication (Gamez et al., 2025). The complexity of their molecular payload, which can include specific combinations of proteins and nucleic acids, and their ability to be selectively taken up by recipient cells, elevates them from simple waste products to pivotal signaling hubs (Marschall, 2021). This transformation in understanding—from passive garbage bags to active, precision-guided communication systems—is a core theme in the field.

**FIGURE 1:**
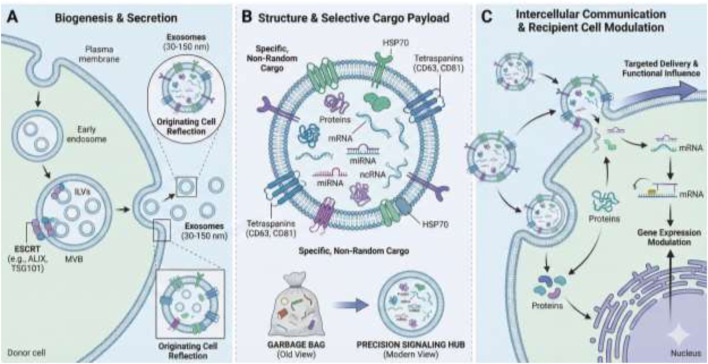
The life cycle and functional shift of exosomes. **(A)** Biogenesis and Secretion The process begins within the donor cell, where the plasma membrane invaginates to form early endosomes. These mature into Multivesicular Bodies (MVBs) containing Intraluminal Vesicles (ILVs). This inward budding is orchestrated by the ESCRT machinery, specifically proteins like ALIX and TSG101, which serve as diagnostic markers. Upon maturation, the MVB fuses with the plasma membrane, releasing the ILVs into the extracellular space as exosomes (typically 30–150 nm in diameter). **(B)** Structure and Selective Cargo Payload Exosomes are encapsulated by a lipid bilayer embedded with canonical markers, including Tetraspanins (CD63, CD81) and heat shock proteins (HSP70). Their internal cargo is a highly specific, non-random “reflection of the originating cell,” containing mRNA, miRNA, ncRNA, and specialized proteins. This panel also highlights the paradigm shift in cell biology: moving from the superseded “Garbage Bag” hypothesis (waste disposal) to the contemporary understanding of exosomes as Precision Signaling Hubs designed for targeted delivery. **(C)** Intercellular Communication and Modulation Released exosomes travel to recipient cells, where they are selectively taken up through membrane fusion or endocytosis. Once inside, the delivered cargo—particularly miRNAs—directly modulates the recipient cell’s functionality by regulating gene expression. This enables even quiescent cells (such as dormant muscle cells) to act as potent signaling sources, maintaining tissue homeostasis and preparing the cellular environment for repair.

Furthermore, the very act of exosome secretion is a regulated signaling event, not a constant process. Research indicates that the number of exosomes released can vary significantly depending on the originating cell’s state (Li H. et al., 2025; Urbanelli et al., 2013; Byun et al., 2021). For example, quiescent muscle cells, which might be considered metabolically dormant, secrete a significantly higher number of exosomes than their actively proliferating counterparts (Li H. et al., 2025; Rocheteau et al., 2014). This finding suggests that a seemingly inactive cell state can be a potent source of signals for tissue homeostasis and preparing for future repair (Rocheteau et al., 2014; Huynh et al., 2019). This challenges a linear view of cellular signaling and instead proposes a more dynamic and nuanced system where even non-proliferative cells contribute to the overall physiological environment (Figure 1).

### Skeletal muscle aging and regeneration: a molecular and cellular perspective

Sarcopenia is not merely a consequence of disuse but a disease driven by a constellation of intrinsic aging processes (Gustafsson and Ulfhake, 2024). It manifests as a loss and atrophy of muscle fibers, particularly fast-twitch type 2 fibers, and a concomitant increase in fat and fibrous tissue (Aversa et al., 2019; Huynh et al., 2019). At the molecular level, sarcopenia is intrinsically linked to two primary processes: the failure to replace senescent cells and an inadequate machinery to maintain cellular and extracellular homeostasis (Gustafsson and Ulfhake, 2024; Pascual-Fernández et al., 2020). Key molecular hallmarks include mitochondrial dysfunction, chronic low-grade inflammation, and disrupted protein homeostasis, characterized by a decline in anabolic signaling (e.g., via mTORC1) and an increase in catabolic activity (e.g., via the ubiquitin-proteasome and autophagy–lysosome pathways) (Damanti et al., 2025; Tu et al., 2025; Aunan et al., 2016).

The regenerative process, particularly following acute injury or chronic atrophy, is fundamentally dependent on the intricate coordination of various cell types, with muscle satellite cells (SCs) serving as the principal, non-redundant stem cell population (Cao et al., 2024; Morisi, 2016). These cells, which reside in a quiescent state beneath the basal lamina of muscle fibers, are activated in response to stimuli like injury or exercise (van de Vlekkert et al., 2020; Abofila et al., 2021). They proliferate and differentiate, either fusing with existing myofibers to add myonuclei (hypertrophy) or forming new muscle fibers to repair damage (Cao et al., 2024; Conceicao et al., 2018). Their function is considered “non-redundant” for muscle regeneration (Magliulo et al., 2021). However, their number and activity decline with age, contributing to the impaired regenerative capacity observed in sarcopenia (Cao et al., 2024; Henrot et al., 2023). Specific miRNAs, such as miR-140-5p, have been shown to modulate SC proliferation and differentiation by targeting key transcription factors like Pax7 (Cao et al., 2024).

Chronic inflammation presents a critical paradox in the context of muscle health. Sarcopenia is linked to this chronic inflammatory state, which can drive muscle breakdown (Damanti et al., 2025; Pérez-Baos et al., 2018). Conversely, exosomes derived from mesenchymal stem cells (MSCs) can promote muscle regeneration by reducing inflammation and promoting the polarization of macrophages from a pro-inflammatory (M1) to an anti-inflammatory (M2) phenotype (Sabaratnam et al., 2022; Arabpour et al., 2021). However, the situation is not always beneficial. Exosomes from inflamed myotubes can induce atrophy and inhibit myogenic signals, suggesting that the context of the source cell is critical (Itokazu et al., 2022). This means that exosomes can act as either pro- or anti-inflammatory agents depending on the health state of the source cell (Harrell et al., 2019). The inflammatory milieu of the aged muscle likely skews exosomal cargo towards pro-atrophic factors, which then contribute to the “molecular scissors gap” and impaired regeneration. This suggests that the quality and composition of exosomal cargo, and not just the presence of exosomes, is paramount in determining their biological effect (Vlassov et al., 2012).

Context dependence is a defining feature of canonical exerkines. IL-6 illustrates this duality: acute, contraction-induced IL-6 release during exercise is linked to anti-inflammatory and metabolic adaptations (including induction of IL-10/IL-1ra and improved substrate handling), whereas chronically elevated IL-6 in metabolic disease and persistent inflammation can associate with catabolic signaling and impaired tissue homeostasis (Severinsen and Pedersen, 2020; Ellingsgaard et al., 2019; Sabzevari Rad and Panahzadeh, 2024; Nara and Watanabe, 2021). Myostatin is similarly pleiotropic: it is a potent negative regulator of muscle mass and repair, yet its network is intertwined with regeneration timing and systemic state; translational programs inhibiting myostatin have shown variable functional benefit, underscoring that blocking a pathway marker does not guarantee clinical impact (Dewasi et al., 2025; Wetzlich et al., 2025; Mitra et al., 2023). IGF-1 generally promotes anabolic signaling and satellite-cell–linked regeneration, but its efficacy is modulated by inflammatory milieu and disease state (e.g., suppressed IGF-1/IGF-1R signaling in chronic conditions), implying that identical EV/exerkine inputs can yield divergent outcomes depending on recipient niche (age, fiber-type composition, insulin resistance) (Mourkioti and Rosenthal, 2005; Tidball and Welc, 2015; Yoshida and Delafontaine, 2020).

While sarcopenia is often described as a progressive, non-reversible decline that is only accelerated by disuse, exercise can significantly improve function in older adults (Gustafsson and Ulfhake, 2024; Sakuma and Yamaguchi, 2018). Given that exercise alters the exosomal cargo—the “exerkine” profile—it is highly plausible that this modulation is a key mechanism by which physical activity intervenes in the pathophysiology of sarcopenia (Safdar and Tarnopolsky, 2018; Bilski et al., 2025). This implies that exercise may not just be a treatment for sarcopenia but a preventative measure, constantly releasing beneficial exosomes to counteract the pro-atrophic signals inherent to the aging process (Ni et al., 2023; Kolodziej et al., 2022).

### The exerkine hypothesis: exercise-induced modulation of exosomal cargo

The concept of skeletal muscle as a secretory organ dates back to the recognition of soluble factors known as “myokines” (Trovato et al., 2019; Mancinelli R. et al., 2021). The “exerkine” hypothesis refines this concept, proposing that many of these humoral factors are released or transported within extracellular vesicles, particularly exosomes, in response to physical activity (Safdar and Tarnopolsky, 2018; Chen et al., 2024) This vesicle-mediated transport provides stability for the cargo, protecting it from degradation in the circulation and allowing for targeted delivery to distant tissues (Cao et al., 2024; Chen et al., 2024).

The effect of exercise on circulating EVs and their cargo is not monolithic; it varies by modality (Maggio et al., 2023). An acute bout of resistance exercise can alter circulating EV size and lead to a relative increase in small EVs, particularly in men (Maggio et al., 2023; Byun et al., 2021). It also causes an increase in circulating exosome-associated proteins (CD63) and a concomitant increase in specific exosomal miRNAs, such as miR-1, in both plasma EVs and recipient adipose tissue (Burke et al., 2024; Bjørge et al., 2018). Long-term resistance training can reduce age-related disparities in exosomal miRNA levels (Ni et al., 2023; Estébanez et al., 2021a). In contrast, acute endurance exercise also increases circulating EVs; while skeletal muscle is a major contributor, multiple tissues likely contribute to the total EV pool *in vivo*, and the net systemic phenotype reflects this mixed-tissue signal (Safdar and Tarnopolsky, 2018; Mastrototaro and Roden, 2024; Wan et al., 2022). Exercise dose (intensity/volume) is an additional, under-discussed modifier of EV release and cargo. Human resistance exercise performed as a high-intensity, whole-body bout has been linked to coordinated changes across muscle, blood EVs, and adipose tissue, supporting the concept that “dose” can influence cross-tissue signal strength (Burke et al., 2024). For example, after high-intensity resistance exercise in humans, skeletal muscle pri-miR-1a increased ∼2.5-fold while EV-associated miR-1 in circulation increased during recovery, consistent with enhanced export of a muscle-derived exerkine signal (Burke et al., 2024). These data provide needed quantitative context that exercise can produce multi-compartment changes beyond “pathway modulation,” supporting physiological relevance. Table 1 summarizes these differential effects.

**TABLE 1:** Differential effects of exercise modalities on exosome profile.

Parameter	Resistance exercise	Endurance exercise	Ref.
Exosome concentration	Increased CD63^+^ EVs	Increased circulating exosomes 1	Byun et al. (2021)
Exosome size	Declined mean size in men, suggesting increase in small EVs	Not specified	Byun et al. (2021)
Protein markers	Increased exosome-associated proteins (CD63^+^)	Enrichment with proteins for mitochondrial biogenesis and fatty acid β-oxidation 3	Byun et al. (2021)
Key miRNA cargo	Increased miR-1 in plasma EVs and adipose tissue 4; differential regulation of miR-378, miR-29a, miR-26a, and miR-451	Not specified	Diez-Roda et al. (2024)
Inter-tissue targets	Adipocytes (via exosomal miR-1) 4	Liver and adipose tissues (via exe-EVs)	Wang et al. (2023)
Modifiers (dose and timing)	Evidence that high-intensity bouts can amplify multi-compartment changes (muscle → EVs → adipose)	Circadian regulation may alter EV release/cargo; timing likely matters	Nederveen et al. (2021)

Definitive “homing” mechanisms for exercise-induced, muscle-derived exosomes remain incompletely resolved. Proof-of-concept from cancer biology demonstrates that EV surface integrins can bias organ tropism and cellular uptake (e.g., specific integrin signatures linked to liver vs. lung uptake), and integrin blockade can reduce uptake in target tissues (Hoshino et al., 2015). Exercise-EV literature supports that EV membrane molecules likely influence uptake routes and tissue interactions, but direct demonstrations that exercise-induced myo-exosomes carry defined integrin codes that drive preferential uptake by osteoblasts or hepatocytes are still limited (Nederveen et al., 2021; Whitham et al., 2018). Accordingly, muscle–liver and muscle–bone axis models should explicitly distinguish between (i) increased circulating EV abundance, (ii) altered cargo composition, and (iii) experimentally validated targeting determinants (surface proteins, integrins, glycan interactions), treating “homing” as a major knowledge gap until systematically tested.

### Beyond muscle: multiple tissues contribute to exercise EVs

Although this review emphasizes muscle-derived exosomes as “exerkines,” exercise acutely mobilizes EVs from diverse sources including adipose tissue, vascular endothelium, immune cells, and platelets, each with distinct surface markers and cargo profiles that can bias tissue targeting and biological outcomes (Nederveen et al., 2021; Burke et al., 2024; Hartwig et al., 2014). This heterogeneity likely explains why the same exercise stimulus can yield different systemic effects across individuals and contexts (e.g., older vs. younger adults; inflamed vs. metabolically healthy states), and it motivates experimental designs that (i) enrich for tissue-specific EVs when possible and (ii) interpret “circulating EV cargo” as a composite signal rather than a pure myo-exosome readout (Nederveen et al., 2021; Moeinfar et al., 2025; Sadri Nahand et al., 2022).

The modulation of specific muscle-associated miRNAs (“myomiRs”) is a key mechanism (Yedigaryan and Sampaolesi, 2021). Exercise modulates the levels of myomiRs such as miR-1, miR-133, miR-206, and miR-486, which are known to revitalize and restore skeletal muscle tissue (Yedigaryan and Sampaolesi, 2021; Vechetti IJ. et al., 2021; Ultimo et al., 2018). The finding that different miRNAs (e.g., miR-378, miR-451) are differentially expressed in high versus low responders to resistance training suggests that the heterogeneity of human response to exercise is not just a function of genetics or training regimen, but is also mediated by a unique exosomal signaling signature (Davidsen et al., 2011). This implies that exosomal profiling could 1 day serve as a diagnostic tool to predict an individual’s response to a specific training program.

A key discovery is the role of exosomal miR-1 in mediating crosstalk between skeletal muscle and adipose tissue (Burke et al., 2024; Guo et al., 2023). An acute bout of resistance exercise leads to increased production of miR-1 in skeletal muscle, which is subsequently released into circulation via EVs (Burke et al., 2024; Csala et al., 2025; Vechetti JrIJ. et al., 2021). These miR-1-enriched exosomes are taken up by adipocytes, where they promote adrenergic signaling and lipolysis by targeting a key gene repressor (Burke et al., 2024; Vechetti JrIJ. et al., 2021; Thompson, 2023). This provides a concrete, mechanistic link between muscle activity and systemic metabolic adaptation. The transfer of this exosomal cargo fundamentally changes our understanding of exercise benefits. The improvements in glucose tolerance and fat metabolism are not simply local, energy-expenditure phenomena but are actively orchestrated by a systemic, vesicle-mediated signaling network originating from the muscle (Crewe, 2023; Bora et al., 2025). This elevates the muscle to a central regulatory organ in whole-body homeostasis, much like the pancreas or liver (Wan et al., 2022; Elliott et al., 2012).

**FIGURE 2:**
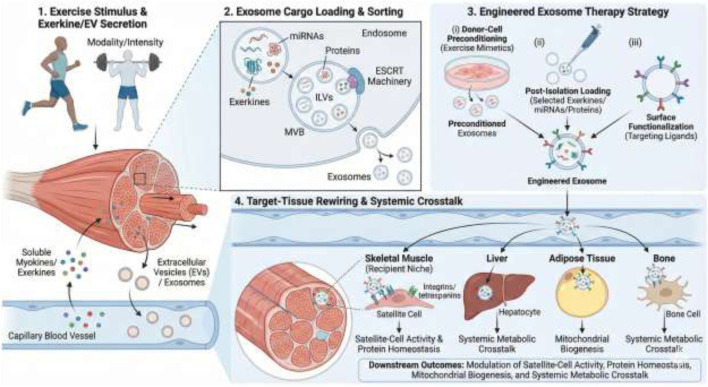
Mechanistic workflow of exercise-induced and engineered exosome therapy for target-tissue rewiring.

This systemic effect also suggests that the exercise-induced exosomal profile may serve as a valuable biomarker for both training status and disease progression. These relationships can be conceptualized as a stepwise pathway linking exercise stimulus, exerkine packaging into extracellular vesicles, and downstream metabolic and regenerative signaling (Figure 2). Since exosomal cargo reflects the health state of the cell of origin, and exercise significantly alters this cargo (Estébanez et al., 2021b), the circulating exosome profile could be a non-invasive way to monitor an individual’s adaptation to training (Vechetti IJ. et al., 2021; Li and Zhanguo, 2025). Furthermore, because aging leads to a decline in beneficial exosomal content, and exercise can mitigate this disparity, exosome profiling could become a biomarker for preclinical sarcopenia or the effectiveness of interventions (Jian et al., 2025) (Table 2).

**TABLE 2:** Key exosomal miRNA cargos and their functions in muscle health.

miRNA	Source cell	Associated stimulus/Condition	Primary target/Pathway	Biological effect	Ref
miR-1	Skeletal muscle	Resistance exercise, mechanical overload	Tfap2α, adrenergic signaling	Promotes adipocyte lipolysis and metabolic adaptations	Burke et al. (2024)
miR-140-5p	Muscle progenitor cells	Muscle regeneration	Pax7	Activates quiescent satellite cells, promotes proliferation and differentiation 7	​
miR-146a-5p	Skeletal muscle	Not specified	Growth Differentiation Factor 5 (GDF5)-PPARγ signaling	Inhibits adipogenesis	Qin et al. (2023)
miR-378	Skeletal muscle	Resistance exercise training	Not specified	Differential regulation in high vs. low responders; positively correlated with muscle mass gains	Diez-Roda et al. (2024)
miR-451	Skeletal muscle	Resistance exercise training	Not specified	Upregulated in low responders only	Diez-Roda et al. (2024)
miR-486-5p	Bone marrow stromal cells (BMSCs)	Dexamethasone-induced atrophy	FOXO1 Axis	Inhibits muscle atrophy	Kargl et al. (2024)
let-7d-3p	Aged adipose tissue	Aging	HMGA2	Blocks muscular stem cell proliferation and contributes to sarcopenia	Jian et al. (2025)
miR-29b-3p	Atrophic myotubes	Senescence, atrophy	Not specified	Associated with pro-atrophic signals	Escudero (2018)
miR-133, miR-206, miR-486	Skeletal muscle	Exercise	Not specified	Revitalize and restore skeletal muscle tissue	Vechetti et al. (2021a)

This figure illustrates the process from exercise stimulus to systemic therapeutic impact. 1. Exercise Stimulus & Exerkine/EV Secretion: Physical exercise (modality/intensity) prompts skeletal muscle to secrete soluble myokines and exerkines, and to release extracellular vesicles (EVs), including exosomes, into the circulation. 2. Exosome Cargo Loading & Sorting: Within the muscle cell, specific cargoes (miRNAs, proteins, exerkines) are sorted into intraluminal vesicles (ILVs) within multivesicular bodies (MVBs) via ESCRT-dependent mechanisms before being released as exosomes. 3. Engineered Exosome Therapy Strategy: Therapeutic exosomes can be generated through three main approaches: (i) donor-cell preconditioning with exercise mimetics, (ii) post-isolation loading with specific therapeutic molecules, and (iii) surface functionalization with targeting ligands to enhance specificity. 4. Target-Tissue Rewiring & Systemic Crosstalk: Following systemic delivery, engineered exosomes are taken up by target tissues. In the skeletal muscle niche, they modulate satellite-cell activity and protein homeostasis. In distant organs like the liver, adipose tissue, and bone, they promote mitochondrial biogenesis and facilitate systemic metabolic crosstalk, leading to widespread beneficial health outcomes.

Timing factors are also likely to modulate exercise-EV biology. Emerging work shows EV biogenesis, release, and cargo composition can be regulated by the circadian clock, implying that identical exercise sessions performed at different times of day may not generate identical EV outputs (Church et al., 2025; Yeung et al., 2022). Nutritional state is a plausible interacting variable because substrate availability and post-exercise feeding alter endocrine and metabolic signaling cascades that intersect with EV loading pathways; however, direct, standardized human studies explicitly manipulating feeding timing/macronutrient composition while tracking EV cargo remain limited, representing a clear gap for future research.

### Exosomal signaling in the muscle microenvironment: from regeneration to degeneration

Exosomes play a direct and potent role in promoting myogenesis. They can stimulate muscle cell proliferation and differentiation, leading to the formation of new skeletal muscle cells and promoting tissue repair and remodeling (Cao et al., 2024; Yue et al., 2020). This is often mediated by the delivery of specific molecular cargos, such as miRNAs that regulate key myogenic pathways like Pax7 and MyoD (Cao et al., 2024; Rahman et al., 2023). For instance, MSC-derived exosomes, in both *in vitro* and *in vivo* models, can significantly enhance muscle regeneration and restore muscle function (Sabaratnam et al., 2022).

However, the therapeutic potential of exosomes is conditional on the context of their source. While MSC-derived exosomes and exercise-induced exosomes are generally pro-regenerative and anti-inflammatory, the presence of a chronic pro-inflammatory state, such as in aging, can be detrimental (Rahman et al., 2023). Exosomes from inflammatory myoblasts can induce inflammation and inhibit myogenic differentiation (Itokazu et al., 2022; Luo et al., 2021). This suggests that the therapeutic potential of exosomes hinges on controlling their source and cargo (Qin et al., 2025). The contradictory findings on inflammatory exosomes and beneficial exosomes underscores a fundamental challenge: exosome therapy is not a one-size-fits-all solution (Zhang et al., 2023). The therapeutic effect depends entirely on the “health” of the donor cell (Wiseman, 2011). Therefore, future therapeutic strategies must not only focus on isolating exosomes but also on engineering them (e.g., loading specific miRNAs or proteins) or carefully selecting their source (e.g., young, healthy donors) to ensure the payload is pro-regenerative rather than pro-atrophic (Jafari et al., 2020).

Critically, recipient competence is a second axis of context dependence. Even a “beneficial” exosome signal may be blunted or redirected in aged, inflamed, insulin-resistant, or fibrotic niches due to altered receptor landscapes, endocytic activity, mitochondrial redox status, and competing cytokine cues (Guo et al., 2023). Thus, future interpretations should treat exosomal effects as an interaction term: (donor/source state × recipient state), rather than attributing outcomes exclusively to donor cargo.

**FIGURE 3:**
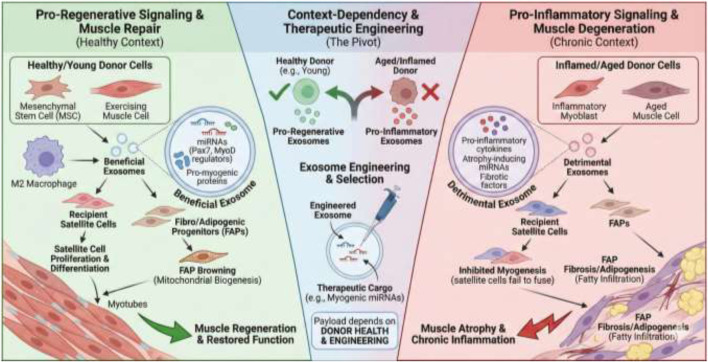
This illustration depicts the context-dependent signaling of exosomes within the multi-cellular muscle microenvironment, demonstrating their capacity to drive either regeneration or degeneration. Exosomes derived from healthy, young, or exercising donor cells (left panel) deliver pro-regenerative cargoes, such as miRNAs regulating key myogenic pathways (e.g., Pax7, MyoD), which stimulate satellite cell proliferation, differentiation, and promote healthy tissue remodeling through interactions with resident cells like macrophages and FAPs. Conversely, exosomes originating from chronically inflamed or aged sources (right panel) carry detrimental payloads of pro-inflammatory cytokines and fibrotic factors that inhibit myogenesis and promote atrophy and fatty infiltration. The central pivot highlights that therapeutic efficacy is not inherent to all exosomes but hinges critically on the “health” of the donor cell, necessitating strategies focused on careful source selection or precise engineering of exosomal cargo to ensure a pro-regenerative outcome.

Furthermore, exosomes facilitate a complex, multi-cellular dialogue in the regenerative niche. The process of muscle repair involves not just satellite cells but also fibroblasts, immune cells (macrophages), and other resident cells (Li H. et al., 2025). Exosomes from quiescent muscle cells can signal to differentiated cells (Liu et al., 2023), and exosomes from M2 macrophages can promote the brown/beige differentiation of fibro/adipogenic progenitors (FAPs), reducing muscle atrophy and fatty infiltration (Xu et al., 2024; Davies et al., 2022). This demonstrates that exosomes are not just acting on muscle fibers themselves but are orchestrating a complex, multi-cellular dialogue within the tissue microenvironment to coordinate the regenerative response. These findings further emphasize that “exercise EV effects on muscle” can arise from non-muscle sources (e.g., immune cell-derived vesicles) that reshape the regenerative niche and thereby alter myogenesis indirectly. This adds a layer of complexity and opportunity for therapeutic intervention beyond just targeting the muscle fiber directly (Figure 3).

### Therapeutic and clinical perspectives: challenges and future directions

Engineered exosomes, with manipulated cargo and surface markers, show promise for enhancing anti-inflammatory, immunomodulatory, and tissue-reparative abilities (Sabaratnam et al., 2022; Ji et al., 2024). For example, MSC-derived exosomes are being explored for their ability to promote muscle regeneration and counteract sarcopenia in preclinical models (Byun et al., 2021; Ji et al., 2024). They have also shown potential for accelerating the healing of sports-related injuries by promoting tissue repair and modulating inflammation (Sabaratnam et al., 2022; Tarnowski et al., 2022). Importantly, several preclinical studies report functional outcomes (e.g., grip strength/endurance readouts, muscle mass ratios, fiber CSA) in addition to pathway markers. For example, in a dexamethasone-induced atrophy model, hUC-MSC-derived exosomes improved performance measures and muscle morphology while simultaneously reducing inflammatory/atrophy markers; notably, the same study quantified systemic endocrine disruption with a 42% reduction in serum estradiol following dexamethasone that was significantly restored by MSC/exosome treatment, illustrating multi-system physiological impact rather than isolated pathway shifts (Li N. et al., 2025).

Despite this promise, significant translational and methodological challenges remain. A major hurdle for clinical translation is the lack of universally accepted protocols for exosome isolation, purification, and characterization (Villarreal-Gómez et al., 2025; Cheng and Kalluri, 2023). Different methods, such as differential centrifugation, size exclusion chromatography (SEC), and commercial kits, have varying advantages and disadvantages, leading to inconsistent yields and purity (Cheng and Kalluri, 2023; Sharma et al., 2024; Barth et al., 1998). The presence of impurities like albumin can “skew the experimental results,” compromising the reliability of research findings (Sharma et al., 2024).

Isolation method strongly shapes what is interpreted as “exerkine-loaded” cargo. Studies included in this review employ heterogeneous EV separation approaches (e.g., ultracentrifugation, precipitation/PEG or polymer kits, ultrafiltration, and size exclusion chromatography [SEC]). These methods differ markedly in purity: precipitation approaches often co-isolate soluble proteins, protein aggregates, and lipoproteins, which can be misclassified as EV cargo and are particularly problematic in plasma/serum where abundant proteins (e.g., albumin) dominate (Kurian et al., 2021; Sidhom et al., 2020). Accordingly, claims that exercise induces “exerkine-loaded exosomes” should, where possible, demonstrate *vesicular encapsulation* rather than co-purification. Consistent with MISEV guidance, rigorous confirmation can include EV marker/negative-marker panels, particle-to-protein metrics, and protection assays (protease/RNase ± detergent) to distinguish intraluminal cargo from surface-adherent or soluble contaminants. When such controls are absent, it is more accurate to describe findings as exercise-modulated extracellular fractions enriched for small EVs rather than definitive exerkine-loaded exosomes (Stahl, 2018).

The regulatory environment also presents a significant barrier. Concerns about the source of human-derived exosomes (e.g., umbilical cords, bone marrow) and the risk of DNA transmission from a diseased donor are paramount (Verma and Arora, 2025; Wu et al., 2025). The lack of a clear regulatory framework from bodies like the U.S. Food and Drug Administration (FDA) has created an unregulated market (Sharma et al., 2024; Aaron, 2023). This regulatory vacuum creates a self-reinforcing cycle of translational failure. Without standardized methods, research results are not reproducible, which in turn hampers the robust data required for regulatory approval (Sharma et al., 2024; Aaron, 2023). This regulatory uncertainty then discourages the large-scale investment needed to develop and commercialize exosome-based therapies, perpetuating the “early stage of research” (Wang et al., 2023).

Most of the current evidence on exosome-based therapies is limited to pre-clinical animal models, with very few clinical trials (Wang et al., 2023; Zheng et al., 2022). This “paucity of clinical evidence” is a major gap that needs to be addressed before widespread adoption (Villarreal-Gómez et al., 2025). Given the risks associated with the source and the potential for a “bad” exosome to spread disease or pathological signals 31, a safer and more precise approach may be the development of synthetic or engineered exosome-mimics (Luo et al., 2024; Villarreal-Gómez et al., 2025). These would bypass the need for human-derived sources, allow for precise control of cargo (e.g., loading specific pro-regenerative miRNAs), and avoid the transmission of unwanted molecules or genetic material (Safadi et al., 2023). This is a crucial future direction for the field. The following table summarizes these key challenges (Table 3).

**TABLE 3:** Methodological and translational challenges in myo-exosome research.

Challenge category	Specific issue	Critical impact on interpretation/Translation	Recommended best practice/Mitigation	References
Isolation & purity	Heterogeneous isolation methods (ultracentrifugation, precipitation kits, SEC, ultrafiltration)	Different methods yield EV preparations with variable purity and composition, making cross-study comparisons unreliable	Clearly report isolation workflow; preferentially combine SEC or density-based separation with particle characterization	Szatanek et al. (2017), Cheng and Kalluri (2023), Sharma et al. (2024)
	Co-isolation of soluble proteins and lipoproteins (especially with precipitation/PEG methods)	Non-vesicular exerkines (e.g., IL-6, IGF-1) may be misinterpreted as exosomal cargo, overstating vesicle-mediated signaling	Avoid precipitation-only claims; interpret results as “EV-enriched fractions” unless vesicular encapsulation is demonstrated	Cheng and Kalluri (2023), Sharma et al. (2024), Barth (1980)
	Insufficient proof of intravesicular cargo	Surface-bound or contaminant proteins/RNAs may confound mechanistic conclusions about “exerkine-loaded exosomes”	Apply MISEV-consistent controls: EV marker + negative marker panels; protease/RNase protection assays (±detergent)	Szatanek et al. (2017), Cheng and Kalluri (2023), Sharma et al. (2024)
Standardization & reproducibility	Inconsistent pre-analytical variables (sample handling, anticoagulants, storage, freeze–thaw cycles)	Alters EV yield, size distribution, and cargo profiles, contributing to poor reproducibility	Standardize collection and handling; report all pre-analytical variables explicitly	Villarreal-Gómez et al. (2025), Cheng and Kalluri (2023)
	Incomplete reporting of particle-to-protein ratios	Inflated protein cargo readouts may reflect contamination rather than EV biology	Report particle counts, protein content, and particle/protein ratios for all EV preparations	Cheng and Kalluri (2023), Sharma et al. (2024)
Biological interpretation	Over-attribution of effects to muscle-derived exosomes	Exercise mobilizes EVs from multiple tissues (adipose, immune cells, endothelium, platelets), confounding source attribution	Use tissue-specific markers or genetic tracing where possible; interpret circulating EVs as mixed-origin	Vechetti et al. (2021b), Safdar and Tarnopolsky (2018)
	Context-dependent exerkine signaling (e.g., IL-6, myostatin, IGF-1)	Identical cargo may produce regenerative or inflammatory outcomes depending on age, metabolic state, or tissue niche	Explicitly define physiological context (acute vs. chronic, young vs. aged, healthy vs. diseased recipients)	Elliott et al. (2012), Safdar and Tarnopolsky (2018) Mancinelli et al. (2021b)
Functional validation	Reliance on pathway modulation without functional outcomes	Changes in signaling proteins may not translate to meaningful muscle regeneration or functional improvement	Include functional endpoints (fiber CSA, strength, endurance, mobility) alongside molecular assays	(Chen et al., 2022; Boppart and Mahmassani, 2019, Wan et al., 2022)
Manufacturing and GMP	Lack of GMP-compatible, scalable production pipelines	Batch variability and undefined potency hinder clinical translation	Develop GMP-aligned workflows with defined release criteria (identity, purity, potency, sterility)	(Jafari et al., 2020; Cheng and Kalluri, 2023; Verma and Arora, 2025)
	Undefined “active ingredient”	EVs lack a single molecular API, complicating regulatory approval	Use mechanism-linked potency assays tailored to intended therapeutic effect	(Jafari et al., 2020; Cheng and Kalluri, 2023)
Clinical translation	Limited biomarker strategies for patient stratification	Heterogeneity of sarcopenia/metabolic disease reduces trial power	Develop and validate circulating EV/miRNA/protein panels for stratification and monitoring	(Estébanez et al., 2021b; Su and Chen, 2025)
	Preclinical–clinical disconnect	Rodent biodistribution, dosing, and immune clearance differ from humans	Incorporate biodistribution, dose–response, durability, and clinically meaningful endpoints early	(Zheng et al., 2022; Wang et al., 2023)
Regulatory & safety	Undefined regulatory framework for exosome therapeutics	Inconsistent oversight risks safety and undermines clinical confidence	Align with evolving FDA/EMA guidance; avoid unregulated human-derived products	(Verma and Arora, 2025; Aaron, 2023)
	Risk of unwanted or pathological cargo	Donor-derived EVs may transmit harmful signals or genetic material	Favor engineered or synthetic exosome-mimics with controlled cargo	( Wiseman, 2011 , Sharma et al., 2024 )

### Translational roadmap for exerkine/exosome therapeutics: GMP manufacturing, patient stratification, and clinical endpoints

Translation requires scalable, validated manufacturing workflows compatible with GMP, including defined release criteria for identity, purity, sterility, safety, and potency, along with batch-to-batch consistency (Cheng and Kalluri, 2023; Andriolo et al., 2023; Wiest and Zubair, 2025). A field-wide barrier is that EVs lack a single “active ingredient,” increasing reliance on orthogonal characterization (marker panels, particle/protein ratios, contaminant profiling) and functional potency assays tailored to the intended mechanism (e.g., pro-myogenic differentiation, anti-inflammatory macrophage polarization) (Wiest and Zubair, 2025; Wiest et al., 2024).

Because sarcopenia and metabolic dysfunction are heterogeneous, biomarker strategies are needed to enrich for responders and interpret outcomes. Emerging work supports circulating biomarker panels (including EV-associated proteins/miRNAs) as candidates for diagnosis and monitoring, but validation remains incomplete and requires standardized pre-analytics aligned with MISEV-type reporting (Aparicio et al., 2024; Lin et al., 2024; Veronesi et al., 2024).

Many EV/exosome studies show pathway modulation *in vitro* or short-term efficacy in rodents, yet clinical translation is limited by species differences in biodistribution, immune clearance, dosing scalability, and clinically meaningful endpoint selection (strength, function, falls, mobility) versus surrogate molecular readouts (Cheng and Kalluri, 2023; Wiest and Zubair, 2025). We therefore recommend that future studies report: (i) dose–response and durability, (ii) biodistribution/uptake assays, (iii) clinically relevant functional endpoints, and (iv) standardized isolation/characterization sufficient to support claims of vesicular cargo.

## Conclusion

The research presented in this review fundamentally redefines our understanding of exercise’s systemic effects. Exercise, far from being a simple mechanical stimulus, acts as a potent biological signal that modulates a complex, multi-organ communication network via exosomes. The “exerkine” hypothesis provides a mechanistic framework for how muscle activity can counteract the molecular hallmarks of aging, promote regeneration, and coordinate metabolic health across the body.

The field stands at a pivotal juncture. While the preclinical evidence for exercise-induced exosomal signaling is compelling, significant challenges remain in translating these findings to clinical practice. The development of robust, standardized methodologies for isolation and characterization is paramount. Future research must not only continue to unravel the specific contents and functions of exercise-induced exosomal cargo but also focus on creating a reproducible and regulated path for their therapeutic application. The promise of harnessing our body’s own communication system to combat muscle aging and disease is immense and represents a transformative frontier in regenerative medicine.
